# Meta-analysis of TSH suppression therapy and the risk of cardiovascular events after thyroid cancer surgery

**DOI:** 10.3389/fendo.2022.991876

**Published:** 2022-12-22

**Authors:** Xiao Yang, Nan Guo, Xin Gao, Jiwang Liang, Xinlong Fan, Yuejiao Zhao

**Affiliations:** Department of Head and Neck Surgery, Liaoning Cancer Hospital & Institute, Shenyang, China

**Keywords:** thyroid cancer surgery, TSH suppression, cardiovascular risk, meta-analysis, differentiated thyroid carcinoma

## Abstract

**Objective:**

To investigate the relationship between TSH suppression therapy and cardiovascular events in patients with thyroid cancer after surgery.

**Methods:**

Pub Med, Web of Science, and Embase databases were retrieved to collect studies related to the risk of cardiovascular events in patients treated with TSH suppression after thyroid cancer surgery. RevMan statistical software was used for meta-analysis.

**Results:**

A total of 14 studies were included. The mean heart rate of patients after thyroid cancer surgery was higher than that of the control group (SMD=2.59, 95% CI: -0.37,.54), and the risk of atrial fibrillation was increased compared with the control group (RR = 1.52, 95%CI, 1.28-1.81; I = 63%). Ejection fraction and left ventricular end-diastolic diameter were not significantly different between the two groups, ejection fraction SMD = -0.10, 95% CI: -3.73, 3.52, left ventricular end-diastolic diameter SMD = -0.09, 95% CI: - 1.29, 1.11. Patients with TSH suppression after thyroid cancer had higher mean systolic blood pressure than controls (SMD = 1.97, 95% CI: −1.09, 5.03) and mean diastolic blood pressure (SMD = 1.85, 95% CI: -0.15, 3.85).

**Conclusion:**

Meta-analysis concluded that TSH suppression therapy after thyroid cancer surgery increases the risk of atrial fibrillation in patients. In addition, the heart rate, systolic blood pressure and diastolic blood pressure are higher than those in the control group, and there is no significant difference in ejection fraction and left ventricular end-diastolic diameter.

## 1 Introduction

Differentiated Thyroid Carcinoma (DTC) includes papillary thyroid carcinoma, follicular thyroid carcinoma, and medullary carcinoma. At present, the standard treatment is mainly surgical treatment, supplemented by postoperative endocrine therapy, radioactive iodine therapy and targeted therapy, and the prognosis is good. Long-term thyroid-stimulating hormone (TSH) suppression therapy is the standard of care after surgery for differentiated thyroid cancer (1). In order to inhibit the secretion of thyrotropin, it is necessary to give a certain dose of physiological dose of thyroxine, of which the most widely used is levothyroxine sodium tablets.

However, exogenous subclinical hyperthyroidism characterized by long-term TSH suppression is thought to be associated with adverse cardiovascular effects including tachyarrhythmias, ischemic heart disease, and hypertension (2, 3). It is worth emphasizing, however, that a cross-sectional study of thyroxine-induced heart disease was observed (4), in which higher than recommended doses of the drug were used. In the presence of intrinsic and pre-existing cardiac abnormalities, associated cardiovascular manifestations may be encountered even in milder degrees of thyroxine overdose. However, in a cohort study of up to 28 years of thyroxine treatment (5), no cardiovascular disease-related changes were found. So far, there is no convincing evidence that taking thyrotropin suppression is harmful to the cardiovascular system.

Therefore, this study intends to conduct a Meta-analysis of cardiovascular adverse events and death risk in patients after TSH inhibition for thyroid cancer surgery by systematically reviewing relevant clinical trials published in recent years, in order to provide a reference for clinical treatment plans.

## 2 Materials and methods

**Figure 1:**
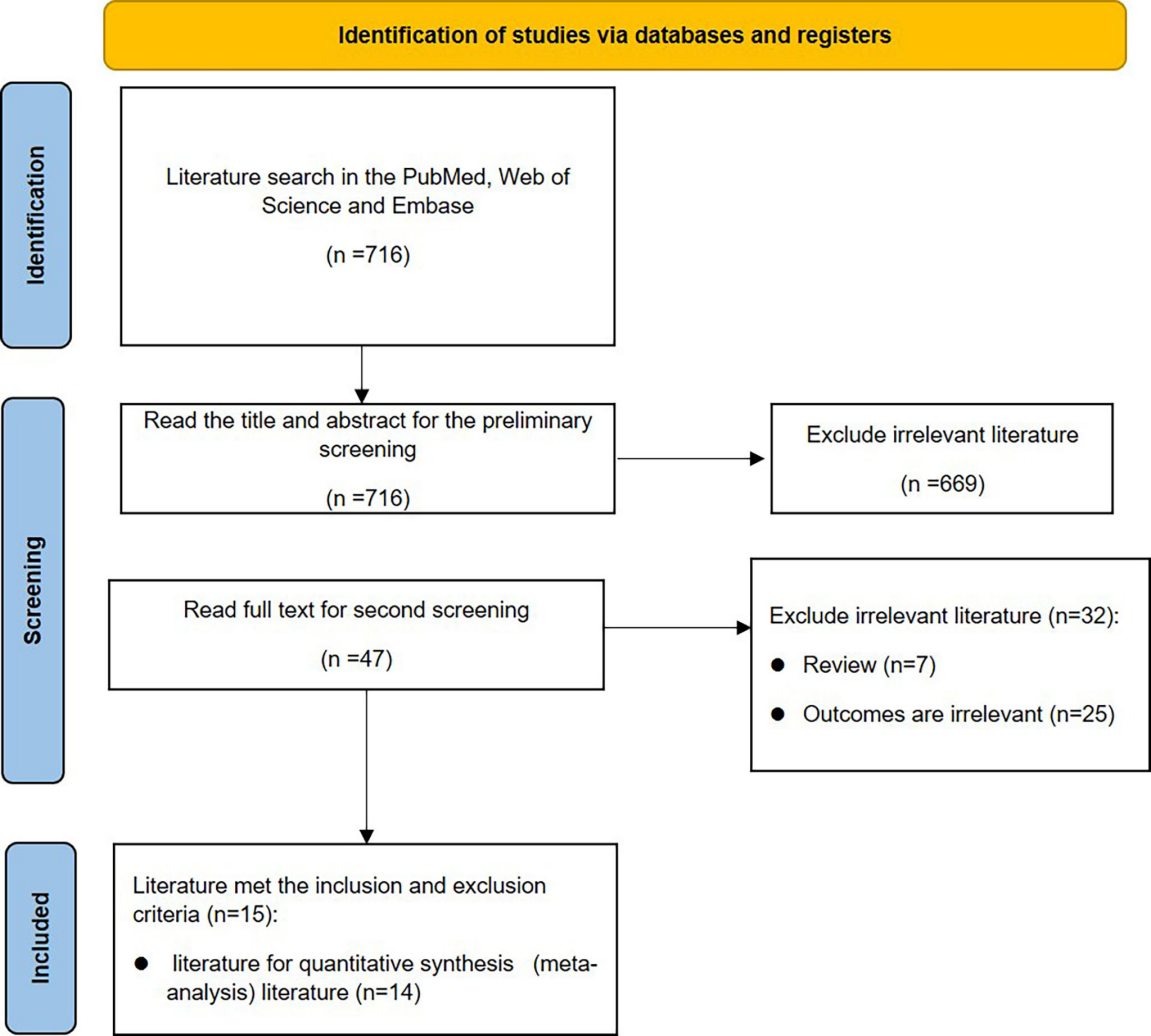
The flow chart of literature screening.

This meta-analysis was conducted in accordance with the PRISMA statement (6). The flowchart is shown in **Figure 1**.

### 2.1 Search process

We conducted a systematic literature search through PubMed, Embase and Web of Science, including all relevant literature from the establishment of the database to May 2022. Search by subject heading combined with free words. The search terms were created using the PICO structure. The key terms are as follows: Patient (P) Thyroid Cancer; Intervention (I) Thyroid hormone suppression after thyroidectomy; Outcomes (O) included cardiovascular disease, arrhythmias, myocardial infarction, ventricular dysfunction, heart failure, and cardiac arrest.

### 2.2 Research options

#### 2.2.1 Inclusion criteria

(1) The original research is a published paper or electronic version; (2) The test method of the type of research should be a case-control study, with complete and reliable data, appropriate statistical methods, and a rigorous control group; (3) Clinical research on the association between TSH suppression after thyroid cancer surgery and the risk of cardiovascular disease; the treatment group was thyroid cancer postoperative patients with cardiovascular disease, the control group is healthy people of the corresponding age group; (4) All patients (case group) have clinical diagnosis of typical cardiovascular disease; (5) Cardiovascular disease is defined as arrhythmia, coronary heart disease, myocardial infarction, essential hypertension, with clear diagnostic and clinical features, such as electrocardiogram, ambulatory blood pressure monitoring, coronary angiography, combined with testing to report abnormal blood lipids, biochemistry, blood pressure or ventricular cardiac function; (6) The included literature provides data on the full TSH inhibitor name and dose.

#### 2.2.2 Exclusion criteria

(1) Case reports, abstracts and reviews, etc.; (2) The experimental design of the original study was not rigorous, or the experimental method did not conform to the case-control study; (3) The diagnostic criteria of the case are not clear; (4) Studies that did not provide the incidence of cardiovascular disease; (5) Documents with incomplete data, and the data could not be obtained after communicating with the author.

### 2.3 Data extraction and quality assessment

Data sets (XX and XX) were extracted from each eligible study by 2 independent reviewers. Required information includes: author name, year of publication, study design, sample size, mean age of subjects, sex, duration of follow-up, experimental and control group interventions, and outcome measures. Meta-analyses were performed only when 3 or more studies reported the outcome of interest. After the above work is completed, cross-check is carried out, and any disagreements will be discussed and resolved under the arbitration of the third author.

### 2.4 Evaluation method

The methodological quality of the included SR was assessed using the AMSTAR 2 tool (7). The tool contains a total of 16 items, including seven key items 2, 4, 7, 9, 11, 13, and 15. It is up to the reviewers to judge whether the included literature meets the requirements of the entry, and then divide the credibility level accordingly. If there are 0 or 1 non-critical items that do not meet the requirements, it is rated as “high confidence”; If more than 1 non-critical item does not meet the requirements, it is rated as “medium confidence”; If 1 key item does not meet the requirements, it is rated as “low confidence”; If more than 1 key item does not meet the requirements, with or without non-critical items, it is rated as “very low confidence”.

### 2.5 Synthesis of evidence

Meta-analysis was performed using RevMan5.4 software to explore the relationship of TSH suppression therapy and the risk of cardiovascular events after thyroid cancer surgery. The continuous data were displayed as mean ± stander division (*X̅* ± S) and the categorical variable was displayed as count. First, the heterogeneity test was performed. If I2 ≤ 50%, the fixed effect model was selected; otherwise, the random effect model was used, and subgroup analysis was performed to find the source of heterogeneity. For continuous variable outcome indicators, the mean difference (MD) or standardized mean difference (SMD) was used to judge the effect size; for dichotomous data, the hazard ratio (RR) was used as the combined effect index, and the 95% confidence interval (CI). A funnel plot was used to test for publication bias. Unless otherwise specified, the test level is set at 0.05.

## 3 Results

### 3.1 The characteristics of included studies

Initially, 714 articles were retrieved. After reading the title abstract, 47 papers were obtained, and the full text was excluded from the review and low-quality literature, and 15 papers were finally included, and 14 studies were performed for quantitative analysis. The search flow chart is shown in **Figure 1**. The publish year of included studies varied 1993-2019, and involved 187,064 patients. The basic characteristics of the included studies are shown in **Table 1**.

**Table 1:** Basic information about TSH suppression and CVD risk after thyroid cancer surgery.

Study	Research design	Sample size(Treat/control)	Gender (M/F)		Age (y)		Follow-up duration (y)
			Treat	control	Treat	control	
Suh et al, 2019 (8)	Cohort	182419:182419	22822/153597	22822/153597	47 ± 11.3	47 ± 11.3	4.3
JoseÍ Botella-Carretero et al, 2004 (9)	Cohort	19:21	Female	Female	42.6 ± 14.1	42.7 ± 14.5	3.8 ± 4.5
Toulis et al, 2019 (10)	Cohort	3009: 11 303	722/2287	2713/8590	50.7 ± 16.1	50.4 ± 15.7	NA
Biondi et al, 1993 (11)	Cohort	20:20	2/8	3/17	36 ± 11	40 ± 9	1-9
Shuvy et al, 2008 (12)	Case-control	20:20	NA	NA	41.9 ± 12.3	41.7 ± 12.2	NA
Ve´ ronique Taillard et al, 2011 (13)	Case-control	24:20	9/34	4/19	54 ± 14	50 ± 11	3 ± 0.9
Hong et al. et al, 2016 (14)	Case-control	14:14	Female	Female	42.9 ± 7.2	42.9 ± 6.5	6.9
Murialdo et al, 2005 (15)	Cohort	11:31	1/10	3/28	56.7 ± 89.4	54.3 ± 15.6	NA
Shargorodsky et al, 2006 (16)	Cohort	26:26	23/3	22/4	52.8	52.6	6.7
Klein Hesselink et al, 2015 (17)	Cohort	518:1563	131/387	372/1164	48.6 ± 14.0	48.6 ± 13.4	8
Nelli Pajamäki et al, 2018 (18)	Cohort	901:4485	171/730	897/3588	48.8 ± 15.9	48.7 ± 15.8	NA
Mittal et al, 2012 (19)	Case-control	50:50	12/38	12/38	58.9 ± 11	57.6 ± 10	NA
Mercuro et al, 2000 (20)	Case-control	19:19	3/16	4/15	44.3 ± 11.8	41.1 ± 11.5	5.7 ± 3.5
Hpftjizer et al, 2009	Case-control	14:24	2/12	4/20	51.6 ± 14.5	45.4 ± 8.5	NA

NA, No specific classification.

### 3.2 The effect of TSH inhibition on the risk of arrhythmia

**Figure 2:**
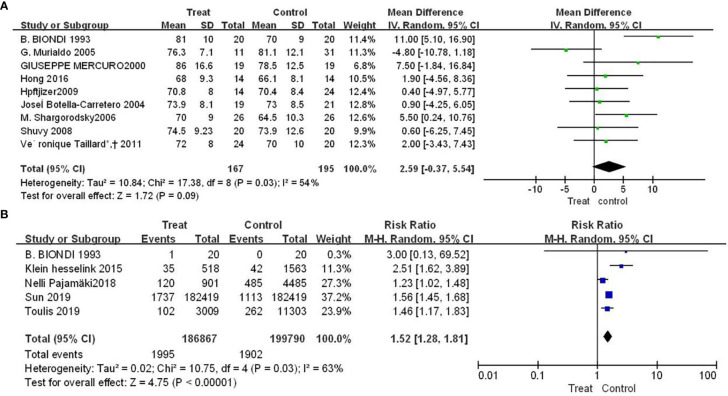
Forest plot of arrhythmia-related adverse events in TSH suppression group and control group, **(A)** heart rate **(B)** atrial fibrillation.

Nine studies showed the effect of TSH inhibition on the risk of arrhythmia. Compared with control group, patients with DTC had a higher mean hear rate (SMD=2.59, 95% CI: -0.37-5.54), but the difference was not statistically significant (P=0.09), shown in **Figure 2A**. The results of **Figure 2B** showed that patients had an increased risk of atrial fibrillation compared with control group (RR = 1.52, 95% CI, 1.28-1.81), and the difference was statistically significant (P<0.00001).

### 3.3 The effect of Euthorac/TSH inhibition on the occurrence of heart failure events

**Figure 3:**
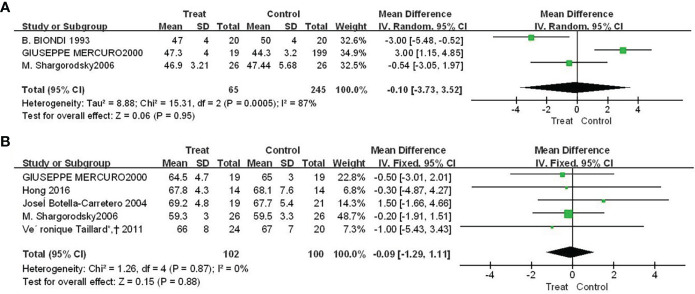
Forest plot comparison of cardiac pumping function between TSH-inhibited group and control group after thyroid cancer surgery. **(A)** ejection fraction, **(B)** left ventricular end-diastolic diameter.

There were three and five studies, respectively, reported the results of Euthorac/TSH inhibition on ejection fraction and left ventricular end-diastolic diameter. The results in **Figure 3** showed that the effect of Euthorac/TSH inhibition on the occurrence of heart failure events were not statistically significant. The effect size of ejection fraction was -0.10 (95%CI: -3.73, 3.52; P=0.95; **Figure 3A**); and the effect size of left ventricular end-diastolic diameter was -0.09 (95% CI: - 1.29, 1.11; P=0.88; **Figure 3B**).

### 3.4 The effect of Euthorac/TSH inhibition on blood pressure

**Figure 4:**
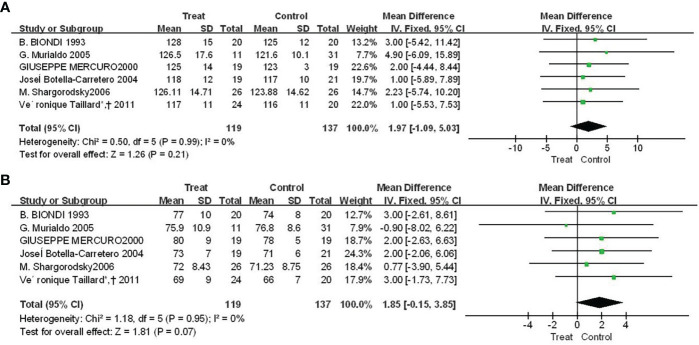
Forest plot of vasoconstriction function between TSH-inhibited group and control group after thyroid cancer surgery. **(A)** Systolic blood pressure, **(B)** Diastolic blood pressure.

There were four studies reported the results of f Euthorac/TSH inhibition on blood pressure. The heterogeneity evaluation results showed a good homogeneity of the included studies, and the fixed effect model was performed. Patients with TSH suppression after thyroid cancer had higher mean systolic blood pressure than controls (SMD = 1.97, 95% CI: −1.09, 5.03) (**Figure 4A**), and mean diastolic blood pressure was higher than Control group (SMD = 1.85, 95% CI: -0.15, 3.85) (**Figure 4B**).

## 4 Discussion

The demerits of this meta-analysis showed that, compared with the control group, patients treated with TSH suppression after thyroid cancer had an increased risk of developing atrial fibrillation, but had no significant changes in ejection fraction and left ventricular end-diastolic diameter. Heart rate, systolic and diastolic blood pressure were higher in patients on suppression therapy.

In this study, the patients had a higher risk of developing atrial fibrillation than the control group and had a faster heart rate. Studies have shown that the effects of thyroid hormones on cardiac chronotropy are manifested as increased risk of tachycardia and atrial fibrillation. Thyroid hormones influence cardiac chronotropy through genomic and nongenomic effects on adrenergic-receptor complex components and sodium, potassium, and calcium channels (21). The basic principle behind thyroid hormone suppression therapy is that when patients take supraphysiological doses of thyroid hormone drugs (such as Euthyrox), the thyroid-stimulating hormone (TSH) secreted by the pituitary gland is suppressed at a low level through negative feedback regulation, so as to inhibit the growth and proliferation of thyroid cancer cells and reduce the recurrence rate of thyroid cancer. Thyroid hormone has the effect of exciting the myocardium, which can increase the heart rate and lead to arrhythmia, as well as increase the myocardial contractility, increase the blood pressure, and increase the cardiac load, leading to the side effects of myocardial hypertrophy and even heart failure. There are obvious related side effects in clinical patients with hyperthyroidism. Both hypothyroidism or hyperthyroidism can accelerate the onset of symptomatic cardiovascular disease. Small changes in thyroxine concentrations may have adverse effects on the cardiovascular system. Subclinical thyroid dysfunction is associated with a 20% to 80% increased risk of vascular morbidity and mortality (22). However, TSH suppression is not equivalent to hyperthyroidism, but little attention has been paid to the relationship between TSH suppression and arrhythmia, and there is a lack of randomized, controlled clinical trials in this field. In future research, not only atrial fibrillation and heart rate, but also other arrhythmia-related events such as life-threatening ventricular fibrillation should be addressed.

Ejection fraction and left ventricular end-diastolic diameter did not change significantly. The myocardium is very sensitive to circulating thyroid hormone levels, changes in thyroid hormone status can lead to heart failure, and heart failure itself can alter thyroid hormone status. Individuals with severe long-term hyperthyroidism can increase cardiac output and further lead to symptoms and signs of heart failure. Patients with episodes of hyperthyroidism have higher long-term cardiovascular mortality due to left ventricular hypertrophy, arrhythmias (eg, atrial fibrillation), and increased cardiac preload secondary to fluid overload (23). However, in a small study of levothyroxine treatment, short- and medium-term effects in patients with dilated cardiomyopathy with biochemically reported normal thyroid levels showed that levothyroxine treatment improved left ventricular ejection fraction and cardiac exercise capacity, and decreased diastolic size and systemic vascular resistance, without causing adverse reactions (24). TSH suppression therapy differs from hormone levels in patients with hyperthyroidism. The choice of existing research methods, the size of the sample, and the length of observation time will all affect the analysis of the results. In the absence of mechanistic studies and large randomized controlled trials, the correlation and causality between the two have not been confirmed.

In this study, compared with the control group, the systolic and diastolic blood pressure of the patients increased to varying degrees. The relationship between clinical thyroid hormone levels and hypertension is unclear. Some patients with hyperthyroidism develop systolic hypertension (25). Several observational studies have shown that subclinical hyperthyroidism is not associated with hypertension (26); However, some studies have shown a positive correlation (27), such as the activation of sodium, potassium, and calcium channels in cardiomyocytes and the systemic vasculature *via* thyroid hormone (21), effects on mitochondrial membranes and mitochondrial production (28), and their relationship to signaling pathways in cardiomyocytes and vascular smooth muscle cells (29). Thyroid hormones activate the PI3K/AKT signaling pathway, induce endothelial nitric oxide production and subsequently reduce systemic vascular resistance (30). Results of a randomized controlled trial showed that levothyroxine treatment improved endothelium-dependent vasodilation in patients with subclinical hypothyroidism (31, 32), although the mechanisms involved are unclear. In addition, the effect of thyroxine on blood pressure is a benefit or a risk, and further research is needed.

Our study has some limitations. First, surgery is the preferred treatment for thyroid and follicular thyroid cancer. However, thyroid and lymph node surgery ranged from conservative to aggressive approaches, which affected the patient’s baseline levels of thyroxine, which in turn affected the effect of TSH suppression on the patient’s hormone levels. In addition, the sample size was small and prospective studies were lacking. Secondly, the data collection of the trial was incomplete, and the trial lacked the records of arrhythmia events of key cardiovascular events. In addition, there were some differences in the collection of some parameters. Ultrasound-related detection was greatly affected by the instrument operator, and many trials lacked this part of the data. Finally, due to differences in baseline and lack of data, TSH levels and cardiovascular adverse events at different doses of TSH inhibitors were not analyzed. Nonetheless, it is necessary to assess the balance of risk for patients with TSH inhibition.

## 5 Conclusion

Meta-analysis concluded that TSH suppression after thyroid cancer surgery increases the risk of cardiovascular events, but more large-scale long-term prospective studies are needed for further study.

## Data availability statement

The original contributions presented in the study are included in the article/supplementary material. Further inquiries can be directed to the corresponding author.

## Author contributions

Conception and design of the research: YZ and XY. Acquisition of data: XY, NG, and XG. Analysis and interpretation of the data: XY, JL, and XF. Statistical analysis: XY and YZ. Obtaining financing: None. Writing of the manuscript: XY and NG. Critical revision of the manuscript for intellectual content: YZ. All authors contributed to the article and approved the submitted version.
